# Building a natural repellent: effects of varying alarm cue exposure on swim activity and spatial avoidance in an invasive fish

**DOI:** 10.1093/conphys/coaf028

**Published:** 2025-04-23

**Authors:** Mikaela E Feder, C Michael Wagner

**Affiliations:** Department of Fisheries and Wildlife, Michigan State University, 480 Wilson Rd., East Lansing, MI 48824, USA

**Keywords:** Behavioural manipulation, invasive species, olfaction, repellent

## Abstract

Techniques for using natural anti-predator cues to guide the movements of animals and reduce human–wildlife conflict are highly desired. With continuous use, sensory adaptation, habituation and adaptive behavioural changes often reduce the efficacy of sensory deterrents. Theory suggests responses can be maintained with application practices that modulate the stimulus in time (on/off) or by continuously varying stimulus intensity. In aquatic environments, damage-released alarm cues from injured conspecifics are a reliable source of information regarding predation risk that can be used to guide the movements of invasive fishes. We used sea lampreys, *Petromyzon marinus*, drawn from an invasive population, to investigate whether modulating alarm cue exposure (on/off) or varying cue concentration during continuous exposure (low/high) would forestall predicted declinations in swim activity and spatial avoidance. We found that continuous exposure to alarm cue at a fixed concentration resulted in the predicted decline in swim activity. Modulating odour exposure timing (on/off) partially prevented response declination in swim activity, whereas varying odour concentration (low/high) fully prevented the reduction. We did not observe the previously reported habituation of the spatial avoidance response, likely due to the use of a small high-throughput assay system. Our results suggest modulating alarm cue exposure by varying odour concentration to prevent response declination holds promise as a management practice. Moreover, test systems for developing management practices should be carefully matched to the scale of the behavioural response being investigated.

## Introduction

Animal repellents are commonly used to reduce human–wildlife conflict. Yet, many suffer from declining efficacy with repeated use [e.g. bears (Elfström *et al*., 2014), birds (Belant **et al*.,* 1993), elephants (O’Connell-Rodwell **et al*.,* 2000), marine mammals (Northridge, 1991), fishes (Dennis and Sorensen, 2020)]. The principal mechanisms underlying reduced efficacy involve physiological (e.g. sensory adaptation, desensitization) and cognitive (habituation) processes, and adaptive behavioural shifts in response to stimuli that are not reinforced by actual risk (Greggor *et al*., 2014, 2020). Any or all may be working against the utility of a particular deterrent. Thus, when a sensory stimulus is used to deter a nuisance species it is important to construct application practices that mitigate these effects through the application of physiological and behavioural knowledge (Blumstein, 2016).

Repellents derived from cues prey use for predator detection tend to elicit more consistent responses over time than other noxious stimuli (Greggor *et al*., 2020). For example, earless seals (Phocidae) prey on salmon raised in offshore net pens causing substantial economic losses (Quick *et al*., 2004). Seals rapidly cease responding to continuous broadcast of cyclic artificial sounds intended to repel them from the net pens (Götz and Janik, 2010). However, the use of a sound optimized to trigger a natural startle and flight response in grey seals (*Halichoerus grypus*), emitted at pseudorandom pulse intervals, achieved a 97% reduction in seal presence over a 19-month period with no evidence of declining response (Götz and Janik, 2016). Similarly, domestic pigs rapidly begin to ignore sound deterrents broadcast continuously, whereas intermittent sound resulted in maintained avoidance (Talling *et al*., 1998). Sound modulation may have worked against the major mechanisms of response declination for at least two reasons. First, sensory adaptation, desensitization and response habituation may be delayed by increasing the time between stimulus pulses (the interstimulus interval or ISI), increasing the intensity of the stimulus or both (Rankin *et al*., 2009). Second, because predation risk varies substantially over space and time, prey frequently update their perception of risk to optimize allocation of time and energy to anti-predator behaviours (Lima, 1998). Paradoxically, the continuous broadcast of a predation-affiliated deterrent may result in animals eventually returning to normal activity in the presence of the unavoidable risk as no time period is perceived as relatively safe (Ferrari *et al*., 2009). Thus, stimulus modulation (e.g. sound volume, odour concentration) may provide an avenue towards more effective broadcast tactics.

The sea lamprey (*Petromyzon marinus*) invaded the upper Laurentian Great Lakes in the late 19th century and has been subject to a pesticide control program operated by the US and Canadian governments since the 1950s (Burkett *et al*., 2021). Although highly effective, additional control methods are desired to address societal desire to reduce pesticide use and to delay the evolution of pesticide resistance (Dunlop *et al*., 2018; Siefkes *et al*., 2021). One promising approach involves the use of an olfactory alarm cue to guide migrating sea lamprey towards traps prior to spawning to limit reproductive success (Hume *et al*., 2015; Hume *et al*., 2020). The ability to guide migrating lampreys would also benefit their conservation. In many places around the world, dams block access to spawning habitat for migratory lamprey species. Although fish passage devices are often installed to promote passage, many suffer from low encounter rates that limit success (Moser *et al*., 2021). Species-specific alarm cues could be used to guide migrants towards these passage opportunities for several species of conservation concern (Byford *et al*., 2016; Porter *et al*., 2017).

Alarm cues are chemical mixtures released from damaged fish tissue after injury that warn nearby conspecifics and closely related taxa about predation risk. Both teleost fishes and lampreys exhibit three classes of olfactory sensory neurons (OSN), including ciliated, microvillous and crypt cells (Hamdani and Døving, 2007; Laframboise *et al*., 2007). The ciliated OSN has been affiliated with response to alarm cue in teleost fishes (Hamdani and Døving, 2003; Dew *et al*., 2014). Which OSN is responsive to alarm cue in lampreys has not been investigated. In sea lamprey, responses to odourants propagate along the olfactory nerve to the olfactory bulb (Green *et al*., 2017). The magnitude of behavioural responses elicited by the odours scale to the odourant concentration for both reproductive pheromones (Li *et al*., 2002) and alarm cue (Wagner *et al*., 2023) suggesting the animal’s perception of opportunities and risks will be affected by physiological and cognitive processes that alter perception.

Exposure to alarm cue elicits antipredator behaviours from fishes and other aquatic organisms including increased vigilance, changes in activity, spatial avoidance and flight (Ferrari *et al*., 2010; Wisenden, 2015). Notably, frequent exposure to heightened predation risk can cause reductions in activity in fishes over spans of hours to days (Hamilton and Heithaus, 2001; Lucon-Xiccato *et al*., 2016) that would harm the ability of managers to guide invasive sea lampreys towards traps in rivers, or native lampreys towards fish passage devices. Over longer periods of exposure (weeks–months), adaptive responses to a persistent high-risk environment should result in reduced anti-predator responses and a return to baseline activity rates (Mirza *et al*., 2006). How sea lamprey activity rates respond to short-term continuous exposure to alarm cue is unknown. However, sea lampreys are known to avoid areas activated with alarm cue (Wagner *et al*., 2011; Bals and Wagner, 2012; DiRocco *et al*., 2016; Wagner *et al*., 2023). In large-arena laboratory tests, this spatial avoidance response attenuates after 4 h of continuous exposure to a fixed concentration of alarm cue, with spontaneous recovery occurring 30–60 min after exposure ends, consistent with habituation (Imre *et al*., 2017; Wagner *et al*., 2022). When the animals were allowed to move in and out of the odour plume at will, the spatial avoidance response was maintained over a 5-h observation period (Wagner *et al*., 2022), suggesting modulating alarm cue exposure timing may forestall reduced efficacy during long-term applications regardless of the underlying mechanism.

Here, our primary objective was to determine whether varying alarm cue exposure timing or concentration would mitigate reductions in swim activity and spatial avoidance in sea lamprey. In the laboratory we examined whether exposure to continuous alarm cue at a fixed concentration for 4 h would (Prediction 1) reduce swim activity in keeping with an adaptive behavioural response and (Prediction 2) reduce or eliminate spatial avoidance of the alarm cue due to habituation. We also tested the effects of modulating alarm cue exposure (on/off) or varying alarm cue concentration (low/high) on (Prediction 3) preserving activity levels and (Prediction 4) eliciting spatial avoidance at levels similar to those observed from unexposed (control) animals. Our secondary objective was to examine the utility of using small-arena testing of single sea lampreys to achieve a high-throughput testing system for studies of odour application practices. We discuss the implications of the results for constructing effective application practices that use olfactory risk cues to manipulate the behaviour of invasive fishes.

## Materials and Methods

### Experimental design

Individual sea lampreys were exposed to one of four conditioning treatments for 4 h prior to behavioural testing: (i) continuous water (control, hereafter labelled water), where sea lampreys were continuously exposed to test arena water; (ii) continuous alarm cue (hereafter labelled AC on), where sea lampreys were continuously exposed to alarm cue at a dilution of 1 μl l^−1^, a dilution that elicits full behavioural reactivity in the laboratory per Wagner *et al*. (2023); (iii) alarm cue pulsed on/off (hereafter labelled AC on/off), where the alarm cue was cycled on/off in 15-min intervals at a dilution of 1 μl l^−1^ and (iv) alarm cue pulsed low/high (hereafter labelled AC low/high), where the animals were exposed to continuous alarm cue with the dilution cycled between low (1 μl l^−1^) and high (10 μl l^−1^) concentration at 15-min intervals. The continuous alarm cue (AC on) is the treatment that elicited loss of the spatial avoidance response in prior studies (Imre *et al*., 2017; Wagner *et al*., 2022).

### Capture of test animals

Adult migratory-phase sea lampreys were captured in June and July of 2020 from traps placed in the Ocqueoc, Cheboygan and Carp Rivers, Michigan, USA. After capture the sea lampreys were transported to the US Geological Survey’s Hammond Bay Biological Station (HBBS) near Millersburg, Michigan, and were held in 1385-l holding tanks receiving a continuous flow of water from Lake Huron. One hundred male sea lamprey that did not exhibit signs of sexual maturity nor external injuries were used in the study. Male and female sea lamprey exhibit similar responses to alarm cue; however, female responses diminish with the onset of sexual maturation (Bals and Wagner, 2012). The test subjects ranged in size from 34 to 54 cm total length (mean ± 1 SD: 46 ± 4 cm) and weighed 72–327 g (mean ± 1 SD: 194 ± 49 g). All animal handling and experimentation procedures were approved by the Michigan State University Institutional Animal Care and Use Committee under permit number AUF 03/18–039-00.

### Alarm Cue collection

Alarm cue was extracted from 81 deceased adult sea lampreys (approximately equal number of males and females) following procedures as described by Bals and Wagner (2012). Euthanized or freshly deceased sea lamprey were kept at −20°C until whole-body alarm cue extraction could take place. Before alarm cue extraction, sea lampreys were thawed, weighed and rinsed with deionized water. Extraction involved a large (2.08 m) Soxhlet extractor fitted with a water-cooled Allihn condenser attached to a 5-l extractor body and a 12-l round bottom flask. Ten litres of 50:50 (weight/weight) 200-proof ethyl alcohol and deionized water were loaded into the flask that was heated by a hemispherical mantle to 75–80°C. Sea lampreys were wrapped in muslin and loaded into the extractor body. The extractor was cycled three times, cooled and stored at −20°C until use. The final concentration of the alarm cue extract was equivalent to 221 mg of tissue per litre of solvent.

### Test apparatus

**Figure 1:**
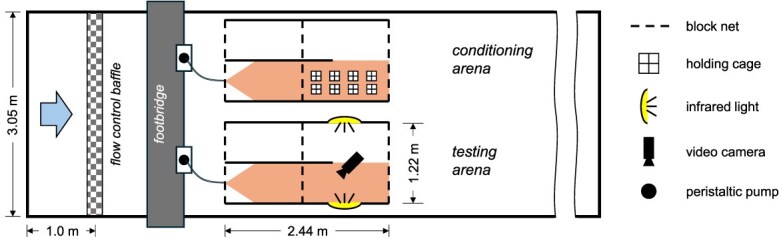
Schematic of the experimental set-up for one of two flumes. The large arrow indicates the direction of flow. Subjects were pre-conditioned in holding cages at the downstream end of the conditioning arena for 4 h. After conditioning a holding cage was slid over to the test arena and the downstream block net replaced. The lamprey was then released. Movement and position in the lower half of the test arena were monitored via overhead video cameras and infrared lights. The conditioning and test odours were introduced with peristaltic pumps.

Trials were conducted in four identical enclosures placed into two large concrete raceways at HBBS (Fig. 1). Each enclosure was fabricated from high-density polyethylene panels and measured 2.44 × 1.22 m (L × W) and was divided with netting into two sections: an upstream mixing zone for odour introduction (1.2 × 1.8 m) and a downstream conditioning or testing arena where the animals were observed (1.2 × 1.8 m). Infrared lights and a video camera (Lorex 8-Channel 4 K UHD NVR with 2 TB HDD and four 5 MP Night Vision Bullets) were installed above the enclosures to record sea lamprey movements. Water from Lake Huron was continuously supplied to the raceways and flowed through the enclosures. Discharge through each raceway was maintained at 0.01 m^3^ s^−1^. During the study period the water temperature ranged from 11.9 to 15.6°C. During conditioning and testing periods, alarm cue was continuously mixed with a stir plate and introduced into the enclosures via peristaltic pumps (MasterFlex model 7533–20).

### Test protocol

Prior to the start of a trial sea lampreys were placed into individual mesh holding cages (30.5 × 30.5 × 15 cm, L × W × D) and placed into the conditioning arena. Each trial consisted of one fish and constituted a single replicate. The prescribed conditioning odour was pumped over the fish for 4 h at a rate of 20 ml min^−1^ via peristaltic pumps. After the 4-h treatment in the conditioning arena, the individual basket was gently moved into the testing arena (submerged) and opened to allow the fish to swim into the centre of the behavioural assay. After the sea lamprey exited the basket it was removed from the arena. The downstream block net was put into place before releasing the sea lamprey. The behavioural test consisted of four consecutive periods totalling 22 min, all of which was video recorded for later behavioural analysis. First, the sea lamprey was allowed to freely explore the test area for 5 min to become acclimated to the conditions. Second, baseline activity was recorded for 5 min including movement rate and time spent on either side of the test area. During the acclimation and baseline recording periods, no alarm cue was present in the arena. Third, alarm cue was introduced into one side of the test area at a rate of 20 ml min^−1^ to achieve a dilution of 1 μl l^−1^ for 2 min. During preliminary trials, 2 min was the time needed for dye plumes to become distributed throughout the half of the test area receiving the alarm cue. The side of the test enclosure receiving the alarm cue was alternated after each trial such that each treatment had an equal number of left-side and right-side applications. Fourth, with alarm cue continuing to be pumped, the behavioural responses to re-exposure to alarm cue (activity rate, proportion of time on the alarm cue side) were recorded for 10 min. All trials were conducted from 19:00 to 02:00 from 1 to 10 July 2020. A total of 24–26 replicates were attempted for each of the four conditioning treatments (water, AC on, AC on/off, AC low/high). We completed 100 trials (water, *n* = 26; AC on, *n* = 24; AC on/off, *n* = 24; AC low/high, *n* = 26). Of these, six were rejected for the water treatment, three for AC on, one for AC on/off and four for AC low/high due to failure to swim into the alarm cue side during the re-exposure period (10 fish), power failures (2 fish) and equipment failures (2 fish).

### Data analysis

To ascertain whether the conditioning treatments affected activity or spatial avoidance of alarm cue, behavioural responses and spatial position were scored using an ethogram (Table 1) and Behavioural Observation Research Interactive Software (BORIS), version 7.9.8 (Friard and Gamba, 2016). These data were recorded from video taken during the baseline activity period (second time period) and during the re-exposure to alarm cue (fourth time period). Videos were scored in a random order and the scorer was blind to the conditioning treatment and the side of the test area receiving the alarm cue. First, each animal’s activity was continuously scored as an integer state variable ranging from 1 to 3, indicating increasing activity from rest/attachment through active swimming. A composite activity score (CAS) for each animal and period was calculated as follows:


$$ \left(\propto \mathrm{AL}1\times 1\right)+\left(\propto \mathrm{AL}2\times 2\right)+\left(\propto \mathrm{AL}3\times 3\right)=\mathrm{CAS} $$

where the CAS represents the sum of the proportion of time (α) in each activity level (AL, 1–3) each multiplied by an individual weighting factor representing increasing activity (AL1, weight = 1; AL2, weight = 2; AL3, weight = 3). The CAS could range from 1 (100% of the time at the lowest activity level) to 3 (100% of the time at the highest activity level).

**Table 1:** Ethogram used to score activity in the test apparatus

Behaviour		Description
State behaviours		
Activity level	1	Sea lamprey is at rest. Attached to the arena with oral disk or laying on the bottom of arena unmoving and unattached
	2	Sea lamprey is actively swimming in the arena at a consistent nominal speed with infrequent turns, without darting; rarely breaches the surface
	3	Sea lamprey increases speed compared to activity level 2. Frequent darting, turns, and breaching of the surface
Frequency behaviours		
Sharp turns		Sea lamprey turns at a ≥90-degree angle in open water
Breaching		Sea lamprey’s head emerges out of water briefly with body vertical in water column

All data analysis was performed using R (R Core Team, 2022). The effect of conditioning treatment on the CAS during the baseline observation period (Predictions 1 and 3) was assessed with a generalized linear model (GLM). As there was a large range in body size, we also investigated whether body size (length or mass) or body condition (g cm^−1^) affected the results. The function ‘glm’ in base R, 4.1.3 (R Core Team, 2022) was used to model CAS. To determine whether activity changed after re-exposure to alarm cue, the CAS measured during the re-exposure period was compared to that measured during the baseline period with a linear mixed-effects model (LMM), function ‘lmer’ under the lme4 package, 1.1–29 (Bates *et al*., 2015).

The number of times either of two behaviours affiliated with increased activity occurred (acute turns of >90° or breaching the water’s surface with the head) were recorded as frequency counts. The rates of expression of each behaviour (events per minute) were calculated for the baseline and re-exposure periods separately. Both breaching and sharp turns were modelled as a function of conditioning treatment using a GLM. Because breaching is thought to be a behaviour affiliated with flight (proposed by Perrault *et al*., 2014), an increased rate of breaching may signify a more ‘fearful’ state. We also performed a linear regression analysis relating the rate of breaching to the composite activity score. We hypothesized a simple linear relationship with non-zero rates of breaching across all activity scores would suggest the number of observed breaches was simply a function of general activity in a confined space and not affiliated with a behavioural state change. The response variable (breaching) was square-root transformed to meet normality (Shapiro–Wilk test, *P* = 0.10) and homoskedasticity (Breusch–Pagan test, *P* = 0.26) assumptions.

All models were analysed using the function ‘Anova’ in the Companion to Applied Regression (car, 3.0-12) package (Fox and Weisber, 2018) to assess for significant parameters. Type II SS was used for models without interactions and Type III SS was used for models with interactions. Following, *post hoc* treatment separation (Tukey's Honestly Significant Difference or Tukey HSD) was conducted using the contrast function in the emmeans package, 1.7.4–1. The *P*-value from the Tukey HSD was used as a measure of strength of evidence against the null hypothesis of no difference versus control, where *P* < 0.05, *P* < 0.10 and *P* > 0.10 indicated substantial, moderate and no evidence, respectively.

To determine whether the conditioning treatments affected the likelihood a sea lamprey would spatially avoid the alarm cue, the position of the sea lamprey in the test arena (alarm cue side or water side) was continuously recorded as a state variable in BORIS during the re-exposure period. The proportion of time spent on the alarm cue side was evaluated with either a one-way *t*-test (passed normality test) or a Wilcox’s rank test (failed normality test) to assess whether the proportion of time spent on the alarm cue side was <50%, indicating avoidance. In the baseline period, we found that the conditioning treatments AC on and AC on/off were not normally distributed (W = −0.85, *P* = 0.004 and W = 0.89, *P* = 0.018, respectively). During the alarm cue re-exposure period, the AC on conditioning treatment was not normally distributed (W = 0.90, *P* = 0.034). If a fish was on the side of the test area without alarm cue during the initial odour introduction (third time period) and remained there for the start of the re-exposure observation period, behavioural scoring did not begin until the animal first entered the alarm cue side. If a fish never entered the alarm cue side of the test area it was censored from the spatial avoidance analysis as it never encountered the alarm (number of censored sea lampreys: water = 4, AC on = 3, AC on/off = 1, AC low/high = 2).

## Results

### Effects of conditioning treatment on activity

**Figure 2:**
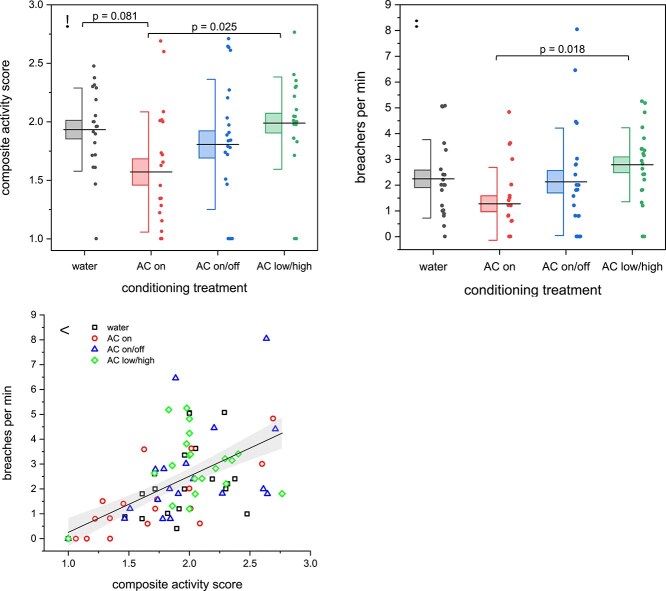
Composite activity scores (**A**) and breaching rate (**B**) observed during testing immediately following conditioning. Horizontal line = mean, box = 1 SE, whisker = 1 SD. *P*-values refer to *post hoc* Tukey HSD tests for contrasts with *P-*values <0.10. Water = control, AC on = continuous alarm cue at fixed concentration, AC on/off = pulsed alarm cue at fixed concentration, AC low/high = continuous alarm cue with varying concentration. (**C**) Breaching rate as a function of the composite activity score across all treatments. Linear fit is to raw data to ease interpretation (shading = 95% CI).

There was substantial evidence that conditioning treatment affected the CAS measured during the baseline period immediately following conditioning (ANOVA, F_86,3_ = 3.20, *P* = 0.028; Fig. 2A). There was moderate evidence that conditioning with 4-h continuous alarm cue at a dilution of 1 μl l^−1^ (AC on) altered activity relative to the water control (*post hoc* Tukey HSD test, *P* = 0.081). Sea lampreys conditioned with continuous alarm cue were 18% less active than those exposed to test arena water (control). Sea lampreys conditioned to AC low/high did not exhibit a reduction in activity relative to the control (*post hoc* Tukey HSD test, *P* = 0.98) and were 21% more active than those conditioned to continuous alarm cue at a fixed concentration (*post hoc* Tukey HSD test, *P* = 0.025). The AC on/off conditioning treatment yielded a mean CAS value that was intermediate to the water and AC on treatments, though there was no evidence it substantially differed from either (water vs AC on/off, Tukey HSD, *P* = 0.9079; AC on vs AC on/off, Tukey HSD *P* = 0.2740). There was no evidence that measures of body size (ANOVA: length, F_86,3_ = 0.32, *P* = 0.57; mass, F_86,3_ = 1.08, *P* = 0.30) or body condition (F_86,3_ = 1.99, *P* = 0.16) affected the CAS score.

Conditioning treatment did not affect the rate of sharp turns observed during the baseline period (ANOVA: F_86,3_ = 0.4188, *P* = 0.74). However, the pattern observed for the number of times sea lampreys breached the water’s surface as a function of the conditioning treatment was similar to that observed for CAS (Fig. 2B). There was evidence that conditioning treatment impacted the observed breaching rates (ANOVA: F_86,3_ = 3.08, *P* = 0.032); yet, only the difference in breaching rate between AC on and AC low/high had support (Tukey HSD, *P* = 0.018). Across all individuals and conditioning treatments, a higher CAS corresponded to a greater frequency of breaching (linear regression, *P* < 0.001, adjusted *R*^2^ = 0.58; Fig. 2C). Only four animals failed to breach during the observation period.

**Figure 3:**
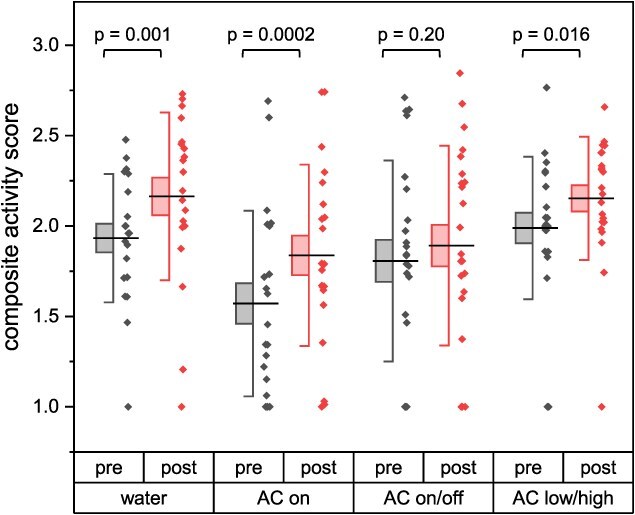
Composite activity scores by conditioning treatment measured after the conditioning period, both before (pre) and after (post) re-exposure to alarm cue in the test flume. Line = mean, box = 1 SE, whisker = 1 SD. *P*-values correspond to *post hoc* Tukey HSD tests following GLM.

There was evidence that re-exposure to the alarm cue after the conditioning period resulted in increased activity for three of the four conditioning treatments (ANOVA: *X^2^*_86,3_ = 7.55, *P* = 0.05; Fig. 3). Sea lamprey conditioned with water increased their activity by 11% (Tukey HSD, *P* = 0.0014). Sea lamprey conditioned with continuous alarm cue at a fixed concentration increased their activity by 14% (Tukey HSD, *P* = 0.0002). Sea lamprey conditioned with continuous alarm cue that varied in concentration increased activity by 7% (Tukey HSD, *P* = 0.016). Sea lamprey conditioned with intermittent alarm did not exhibit a change in activity (Tukey HSD, *P* = 0.20).

### Effects of conditioning treatment on spatial avoidance of alarm cue

We observed no evidence of preference for one side of the test arena during the baseline monitoring period following conditioning for any of the treatments. All tests did not differ from a 50:50 distribution (water, t_19_ = 1.5, μ = 0.50, *P* = 0.92; continuous alarm cue at fixed concentration, Wilcoxon’s rank test_20_ = 169.5, μ = 0.50, *P* = 0.97; continuous alarm cue with varying concentration, t_21_ = −0.18, μ = 0.50, *P* = 0.43; intermittent alarm cue, Wilcoxon’s rank test_22_ = 138, μ = 0.50, *P* = 0.51). After re-exposure to the alarm cue, the proportion of time spent on the alarm cue side did not differ from 50% for any conditioning treatment (water, t_19_ = 0.53, μ = 0.50, *P* = 0.69; continuous alarm cue fixed concentration, t_21_ = 0.06, μ = 0.50, *P* = 0.53; intermittent alarm cue, t_22_ = 0.53, μ = 0.50, *P* = 0.69).

## Discussion

As predicted (Prediction 1) we observed evidence of a substantial reduction in baseline swim activity related to conditioning with continuous alarm cue at a fixed concentration (vs the water control), the treatment that elicited loss of the spatial avoidance response in prior studies (Imre *et al*., 2017; Wagner *et al*., 2022). It is likely this outcome was related to adaptive changes in behaviour.

Exposure to alarm cue in fishes induces a range of anti-predator responses that affect movement including area evacuation, increased activity, freezing or decreased activity and shelter-seeking (Ferrari *et al*., 2010; Wisenden, 2015). The tactic chosen is often responsive to the circumstance (e.g. shelter-seeking when shelter is near, freezing when it is not) and reflective of recent experience. For example, cichlids exposed to high-frequency alarm cue pulses over 3 days exhibited a lower alarm response to most concentrations of alarm cue than those infrequently exposed, with both groups exhibiting overall reduced activity rates (Brown *et al*., 2006). Here, sea lampreys were conditioned to alarm cue in relatively small holding cages, potentially simulating shelter, and then released into a novel arena after brief handling. Sea lampreys continuously exposed to alarm cue may have adjusted their behaviour in recognition of occupation of a high-risk environment, resulting in reduced activity. The reduction in baseline swim activity may also have been affiliated with the induction of exploration neophobia, a known consequence of exposure to alarm cue in fishes (e.g. Abudayah and Mathis, 2016; Brown *et al*., 2016; McCormick *et al*., 2017). However, we may expect that the continuous alarm cue conditioning with varying odour concentration would have simulated the greatest overall risk—a persistent background risk coupled with periods of high risk—resulting in greater reductions in activity and exploration neophobia. That did not occur. Within an adaptive paradigm, this could be related to overall risk, where the continuous low background conditioning treatment represented a moderate risk eliciting reduced activity (and increased vigilance), whereas the low/high conditioning treatment represented alternating periods of low and high risk, causing the animals to attempt escape from the area when the cue was absent (i.e. during the initial observation period), consistent with adaptive decision-making (Lima, 1998; Ferrari *et al*., 2009).

Importantly, we did observe evidence that modulating the alarm cue resulted in maintenance of activity levels akin to those observed in animals that were conditioned with water (Predictions 3 and 4). The intermittent-exposure treatment (on/off, Prediction 3) partially mitigated the declination in swim activity, though there was no statistical evidence that the difference was meaningful. However, there was strong evidence that alternating the alarm cue concentration low/high maintained the activity rate observed in the control animals. These findings suggest either approach is likely to improve the performance of alarm cue applied as a repellent, with the varying concentration approach working the best. That approach is likely to be the method preferred by environmental managers for guiding migrating lampreys towards fish passage devices or traps, depending on the population of interest, as it avoids periods without the repelling stimulus and should maximize encounter rates with the devices. In the Great Lakes, low encounter rate is the factor most responsible for underperforming traps (Bravener and McLaughlin, 2013; Rous *et al*., 2017).

Despite the prior reports (Imre *et al*., 2017; Wagner *et al*., 2022), we did not observe evidence in support of habituation of the increased activity or spatial avoidance responses to alarm cue (Prediction 2) when sea lampreys were exposed to continuous alarm cue at a fixed concentration for 4 h. Habituation is a rich neurophysiological process that alters how an animal responds to a stimulus when repeatedly or continuously exposed (Rankin *et al*., 2009). Although typified by reductions in the expression of certain behavioural responses over time, not all aspects of a response will habituate at the same rate or to the same extent, as each component may be underlain by distinct physiological mechanisms (Rankin and Broster, 1992; McDiarmid *et al*., 2019). Thus, habituation of each component of a behavioural response should be evaluated separately (here, activity and spatial avoidance).

Both control- (conditioned with water) and alarm cue-conditioned sea lampreys exhibited an increase in activity upon re-exposure to the cue in the second observation period. This indicates: (i) the olfactory epithelium continued to respond to the cue through increased electrical activity, precluding sensory adaptation or desensitization and (ii) they exhibited a behavioural response, precluding habituation. Notably, sea lampreys conditioned to the fixed concentration of alarm cue did not recover to the full activity exhibited by the control animals after re-exposure to alarm cue, suggesting their perception of the risk associated with that odour concentration had shifted downward. Sea lamprey exhibit threat-sensitive responses to alarm cue, escalating response intensity as the stimulus concentration increases (Wagner *et al*., 2023). An effect of habituation is to reduce an animal’s perception of the threat associated with a particular stimulus intensity (Rankin *et al*., 2009). Consequently, sea lamprey may have been habituating, but not fully habituated, thereby perceiving a lower absolute threat level. However, they would have experienced the same incremental increase in threat when re-exposed to alarm cue (vs the control animals), causing them to show a similar magnitude of increase in activity, but not a similar activity rate. We think this is less likely than the adaptive behavioural response explanation, but cannot preclude it as a contributor to the observed responses.

We also did not observe evidence of the predicted alarm cue habituation of the spatial avoidance response regardless of the alarm cue conditioning treatment, nor the expected spatial avoidance of alarm cue in the control animals. We suspect this was due to an inappropriately small test chamber. A small-arena test may be inadequate for measuring spatial responses in a highly active fish that is engaged in a large-scale upstream migration and is perceiving immediate risk of predation. Although Mensch *et al*. (2022a) detected avoidance of putrescine by sea lamprey in an identical arena, the effect size was relatively small. In the presence of an anti-predator cue, escape may have become paramount and the fish explored all possible routes out of the test arena. Notably, the rate of water surface breaching was correlated with overall activity. As such, the data does not support the conclusion that breaching is a distinct behaviour associated with flight. Sea lampreys are observed exposing themselves on wetted stream banks and scrambling over rocks when passing through very shallow riffles during the spawning migration (Applegate, 1950). The breaches we observed were typically along the sides of the tank and likely were related to attempts to exit the confines of the arena.

It is also plausible that small test arenas can both decrease the organisms’ perception of safety (Mackay *et al*., 2021) and induce stress with confinement (Sanches *et al*., 2015; Lawrence *et al*., 2018). It has been recommended that behavioural arenas should be 4–15 times their body width and length (Jutfelt *et al*., 2017). The behavioural assay used in this study was 2–3 times larger than the body length of the sea lamprey with a shallow water depth. Migrating lampreys are thought to avoid the shallow margins of rivers where they are vulnerable to nocturnal shoreline predators (Cochran, 2009; Kerr *et al*., 2023; Griffin *et al*., 2025). In a recent study of migrating sea lampreys ascending an eel ladder fish passage device, prior exposure to alarm cue made them less likely to attempt passage, a behaviour consistent with risk-averse decision-making (Hume *et al*., 2020). In our study sea lampreys consistently exhibited increased activity when re-exposed to alarm cue in the small arena tests, indicating they continued to detect and respond to the cue after the conditioning period regardless of the conditioning treatment. We therefore cannot offer any conclusions as to whether the spatial avoidance response was habituated in the AC on treated fish, nor whether the modulated alarm cue treatments (AC on/off or AC low/high) would prevent habituation of the spatial avoidance response. We can recommend against the use of small test arenas to make that determination. Whether the low/high alarm cue application approach would also forestall or eliminate habituation of the spatial avoidance response merits further testing with a return to large-arena approaches. Although this will result in slower discovery (i.e. the approach is not high throughput), the results are likely to be more reliable.

## Conclusions

The use of a natural odour to repel an animal requires application practices that prevent behavioural response declination with repeated exposure (Blumstein, 2016; Greggor *et al*., 2020). The results obtained from our examination of methods to reduce response declination to a natural antipredator cue in sea lamprey suggest effective deterrent application practices are possible but require careful development in appropriate test circumstances. Applying alarm cue with time-varying changes in concentration fully mitigated changes in swim activity associated with continuous exposure to alarm cue, and may prove the most effective application in conservation activities. However, our failure to elicit a spatial avoidance response in the small-arena tests necessitates further investigation under field conditions, as spatial avoidance is the most desired response and different behavioural responses to a single stimulus may habituate at different rates (McDiarmid *et al*., 2019). In the field, we may also expect to observe the effects of freedom to move into areas of the river not activated by the alarm cue, further reducing the likelihood of habituation (Wagner *et al*., 2022). The swim activity response is likely to prove very valuable in small-arena high-throughput testing to discover the chemical constituents of the sea lamprey alarm cue (Mensch *et al*., 2022b).

## Supplementary Material

Web_Material_coaf028

## Data Availability

All data used in the analyses are included as an Excel spreadsheet in the Supplemental Materials.
